# A breath of fresh air: targeted non-viral in vivo gene correction in the mammalian lung

**DOI:** 10.1038/s41392-024-01994-y

**Published:** 2024-10-14

**Authors:** Jixin Liu, Dirk Grimm

**Affiliations:** 1 Department of Infectious Diseases/Virology, Section Viral Vector Technologies, Medical Faculty, University of Heidelberg, BioQuant, Center for Integrative Infectious Diseases (CIID), 69120 Heidelberg, Germany; 2 German Center for Infection Research (DZIF) and German Center for Cardiovascular Research (DZHK), partner site Heidelberg, 69120 Heidelberg, Germany; 3 Faculty of Engineering Sciences, University of Heidelberg, 69120 Heidelberg, Germany

**Keywords:** Gene delivery, Genetic engineering

**Fig. 1:**
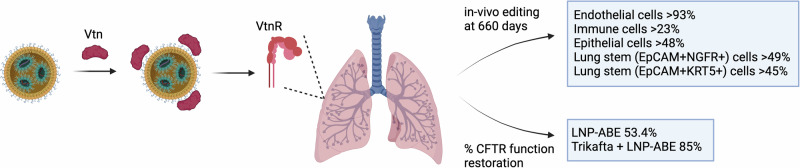
Lipid nanoparticles (LNPs) bind to vitronectin, which facilitates their uptake by vitronectin receptors (VtnR) in the lungs. The figure illustrates the efficiency of gene editing in various lung cell types and the restoration of CFTR function. This figure was created with BioRender

In a recent study published in *Science*,^1^ Sun and colleagues showcase the power and potential of lung SORT LNPs, *i.e*., lipid nanoparticles that upon systemic delivery in mice specifically and efficiently target cells in the lung, most likely facilitated by their binding to plasma vitronectin and uptake via the vitronectin receptor. Most remarkably, when engineered to deliver a base editor, peripheral injection of SORT LNPs enabled highly efficient gene correction in lung stem cells, whole lung and trachea in a mouse model of cystic fibrosis, illustrating the enormous promise of this novel technology for human patients suffering from this devastating disease (Fig. 1).

As one of the most cutting-edge therapeutic approaches, gene therapy has been in the scientific spotlight for decades. It brings hope for numerous acquired or inherited disorders with unmet medical needs, by enabling the correction of the underlying genetic defects with a single drug administration. Key to success is the delivery of the gene therapeutic, which aims to precisely target the diseased tissues or cells, and to efficiently and safely release the therapeutic cargo. Currently, gene therapy mainly relies on either viral or non-viral delivery approaches. Among these, recombinant Adeno-associated viruses (AAV) are the leading viral gene delivery vehicle (or vector), while the most promising non-viral delivery vehicles are lipid nanoparticles (LNPs). Unlike AAVs or other viral vectors that have a natural tropism, non-engineered LNPs have little to no tissue or cell specificity and therefore primarily end up in the liver following systemic delivery. Importantly, though, recent advances in LNP engineering for tissue-specific delivery have shown exciting outcomes for various diseases and targets, especially in differentiated cells such as liver and immune cells.^2^

The study by Sun et al.^1^ capitalized on these advances and aimed to mediate gene editing in cells with lung SORT LNPs, with the goal to avoid loss of exogenously introduced genome editing machinery during cellular differentiation. Intriguingly, the in vivo use of lung SORT LNPs to deliver the mRNA of Cre recombinase enabled long-term expression in mice in edited lung stem cells that lasted for over 1.8 years. Flow cytometry analysis indicated that, besides lung stem cells, endothelial cells, epithelial cells, and immune cells also reached high editing efficiency. One week after injection, more than half of all lung cells expressed the reporter gene for 660 days. At this end point, almost all of the endothelial cells, a majority of the epithelial cells and a moderate fraction of the immune cells were successfully edited. Lung stem cells, as identified by two different nerve growth factors, retained most of the initial expression levels throughout the study. Additionally, lung SORT LNPs were also used to deliver CRISPR-Cas9, with the expression lasting for over eight months in the lungs and without affecting kidney or liver function. Curiously, most lung-resident immune cells (other than macrophages) and lung endothelial cells have a short life span and a high turnover rate ranging from a few days to several weeks, making the authors wonder how those lung cells managed to maintain long-term expression in their study. Sun and colleagues subsequently found that endothelial progenitor cells and lung-resident hematopoietic stem or progenitor cells show high editing efficiency, explaining the persistence of gene editing in mature cells. Next, the authors explored the mechanism of lung-specific targeting by assessing the cellular uptake machinery. Interestingly, this revealed that the SORT LNPs bind a plasma protein called vitronectin (Vtn),^3^ which can facilitate their uptake in cells expressing the vitronectin receptor (VtnR). In line with this, the lung was found to be much more VtnR-enriched compared to all other organs, which strongly supports the role of VtnR in mediating the lung-specific uptake of lung SORT LNPs.

Motivated by this encouraging data, the lung SORT LNPs were further assessed for their therapeutic potential for treatment of cystic fibrosis (CF), a genetic lung disease caused by a C-to-T mutation in the cystic fibrosis transmembrane conductance regulator (CFTR). To this end, the authors applied a published therapeutic strategy^4^ to deliver adenine base editor (ABE) mRNA together with single-guide RNA sgR553X to correct the nonsense mutation in primary and immortalized human bronchial epithelial (HBE) cells. Notably, LNP-ABE achieved near complete correction in 16HBEge CFTR-R553X cells. In primary HBE cells derived from CF patients with the R553X-F508del mutation, LNP-ABE achieved substantial allelic correction in both basal cells and differentiated cells. Sequencing and analysis confirmed a pronounced editing frequency at the T_7_ position (desired), versus a more moderate rate at the T_11_ position (a silent mutation) and no editing at the T_17_ position. Capillary Western blotting showed that LNP-ABE doubled the amount of core glycosylated CFTR and improved the levels of fully glycosylated mature CFTR by more than five-fold. Combining the current standard-of-care treatment Trikafta (for patients with the F508del mutation) with LNP-ABE additionally boosted these levels by over seven-fold. Moreover, the activity of CFTR-dependent chloride ion channels was measured to assess the restoration of HBE function. Compared to the HBE function in healthy donors, LNP-ABE alone was able to restore more than half of the CFTR function, with even greater restoration observed when combined with Trikafta. This is remarkable considering that the therapeutic threshold is 10% restoration of CFTR function. Hence, these results imply that lung SORT LNP-ABE can restore CFTR function by effectively correcting undifferentiated lung basal cells and producing mature epithelium.

Lastly, the LNP-ABE was evaluated in vivo in a CF mouse model harboring the whole human exon 12 that contains the R553X mutation. Notably, as this model lacks the pathological phenotypes typically observed in the lungs of human CF patients, mouse intestinal stem cells were used to generate organoids. After treatment with LNP-ABE, most organoids swelled, indicative of elevated CFTR activity, and nearly 50% sequence correction was confirmed. Furthermore, heterozygous mice that carry the humanized R553X mutation in one allele and the normal mouse CFTR in the other were used to assess base editing efficiency. Systemic administration of LNP-ABE corrected half of the T_7_ position in lung stem cells, while moderate correction was detected in whole lung and trachea. These final results of the study are most notable, as they suggest effective in vivo correction of mouse lung stem cells with LNP-ABE from a clinically pertinent peripheral delivery route.

Collectively, this study demonstrates the great capacity of lung SORT LNPs to specifically and efficiently target various lung cell types in vivo. Moreover, it sheds light on the mechanism underlying the observed tissue specificity, by identifying the critical role of Vtn and its receptor VtnR for LNP uptake. Finally, this work impressively exemplifies the therapeutic potential of this novel technology in primary human CF models and in vivo CF mouse models, providing substantial optimism and excitement for its translation in human patients suffering from this largely intractable disease.
